# Discussion on the case from neurodevelopmental and transdiagnostic perspectives

**DOI:** 10.1192/j.eurpsy.2025.201

**Published:** 2025-08-26

**Authors:** A. Lingford-Hughes

**Affiliations:** Psychiatry, Imperial College London, London, United Kingdom

## Abstract

**Abstract:**

During the workshop about this complex case, I will discuss aspects of the case and the challenges the individual has faced from a neurodevelopmental and transdiagnostic perspective. It is the norm that those presenting for treatment or support with problematic drug/alcohol use will also have co-occurring neurodevelopmental, mental and physical health traits or disorders. Their relationships are often complex and ingrained. It is generally unhelpful to consider such complexity in terms of **‘**primary’ vs **‘**secondary’ as this may lead to exclusion from some services. For instance, someone with depression and problematic alcohol use may not be seen by psychiatric services or psychologists due to their drinking and an **‘**addiction’ service may not have sufficient mental health expertise for managing their depression. Such **‘**silos’ are common and need to be addressed through better understanding of the relationships between problematic drug/alcohol use and co-occurring neurodevelopmental, mental and physical health traits or disorders, through better training of health and social care staff and reducing stigma.

**Disclosure of Interest:**

A. Lingford-Hughes Grant / Research support from: Research supported by Lundbeck, GSK, Indivior; unrestricted funds support from Alcarelle for a PhD, Consultant of: Silence, NET Device Corps, Sanofi-Aventis, Astra Zeneca and also consulted by but received no monies from Britannia Pharmaceuticals, GLG, Opiant, Lightlake and Dobrin, Paid Instructor of: Received Honoraria paid into her Institutional funds for speaking and Chairing engagements from Lundbeck, Lundbeck Institute UK, Janssen-Cilag, Pfizer, Servier; receives Honoraria for teaching for British Association for Psychopharmacology.

